# Body Mass Index From Age 15 Years Onwards and Muscle Mass, Strength, and Quality in Early Old Age: Findings From the MRC National Survey of Health and Development

**DOI:** 10.1093/gerona/glu039

**Published:** 2014-03-28

**Authors:** Rachel Cooper, Rebecca Hardy, David Bann, Avan Aihie Sayer, Kate A. Ward, Judith E. Adams, Diana Kuh

**Affiliations:** ^1^MRC Unit for Lifelong Health and Ageing at UCL, UK.; ^2^Academic Geriatric Medicine, MRC Lifecourse Epidemiology Unit, University of Southampton, UK.; ^3^MRC Human Nutrition Research, Elsie Widdowson Laboratory, Cambridge, UK.; ^4^Clinical Radiology and Academic Health Science Centre, Manchester Royal Infirmary, Central Manchester University Hospital NHS Foundation Trust, UK.

**Keywords:** Sarcopenia, Obesity, Life course epidemiology, Muscle mass, Strength and quality.

## Abstract

**Background.:**

As more people live more of their lives obese, it is unclear what impact this will have on muscle mass, strength, and quality. We aimed to examine the associations of body mass index (BMI) from age 15 years onwards with low muscle mass, strength, and quality in early old age.

**Methods.:**

A total of 1,511 men and women from a British birth cohort study with BMI measured at 15, 20, 26, 36, 43, 53, and 60–64 years and dual-energy x-ray absorptiometry scans at 60–64 years were included. Four binary outcomes identified those in the bottom sex-specific 20% of (a) appendicular lean mass (ALM) index (kilogram per square meter), (b) ALM residuals (derived from sex-specific models in which ALM (kilogram) = β_0_ + β_1_ height [meter] + β_2_ fat mass [kilogram]), (c) grip strength (kilogram), (d) muscle quality (grip strength [kilogram]/arm lean mass [kilogram]). Associations of BMI with each outcome were tested.

**Results.:**

Higher BMI from age 15 years was associated with lower odds of low ALM but higher odds of low muscle quality (per 1 *SD* increase in BMI at 36 years, odds ratio of low ALM residuals = 0.50 [95% CI: 0.43, 0.59], and muscle quality = 1.50 [1.29, 1.75]). Greater gains in BMI were associated with lower odds of low ALM index but higher odds of low muscle quality. BMI was not associated with grip strength.

**Conclusions.:**

Given increases in the global prevalence of obesity, cross-cohort comparisons of sarcopenia need to consider our findings that greater gains in BMI are associated with higher muscle mass but not with grip strength and therefore with lower muscle quality.

Detrimental age-related changes to the structure and function of muscle that typically occur from midlife onwards even in the absence of disease are well documented (1–3) and have important implications, especially in the context of global population aging. For example, poor muscle structure and function are strongly associated with subsequent health and survival (4,5) and with high estimated health care costs (6).

Although existing evidence shows cross-sectional associations of contemporaneous body mass index (BMI) and fat mass with muscle mass, strength, and quality (7–12), there are very few existing studies relating earlier measures of adiposity to subsequent measures of muscle (13,14). With the increasing prevalence of overweight and obesity worldwide, growing numbers of people are reaching old age having lived more of their lives overweight or obese. Identifying the likely impact of prolonged exposure to high BMI and to changes in BMI in different periods of adulthood on subsequent muscle mass, strength, and quality in later life is thus important.

Using prospective data from a nationally representative sample of British people in early old age, we compared the associations of BMI from age 15 onwards and BMI gain in earlier and later adulthood with four different measures of muscle: muscle mass (with and without adjustment for fat mass), strength, and quality.

## Materials and Methods

### Study Population

The Medical Research Council National Survey of Health and Development (NSHD) is a socially stratified sample of 5,362 singleton births that took place in 1 week of March 1946 in mainland Britain. Between 2006 and 2010 (at 60–64 years), 2,856 eligible study members (those known to be alive and living in England, Scotland, or Wales) were invited for an assessment at one of six clinical research facilities (CRFs) or to be visited by a research nurse at home of whom 2,229 were assessed (1,690 at a CRF) (15,16).

Relevant ethical approval was obtained, and participants provided written informed consent.

### Muscle Characteristics and Fat Mass at 60–64 Years

Measures of body composition were obtained for 1,658 (98%) CRF participants in the supine position using a QDR 4500 Discovery dual-energy x-ray absorptiometry (DXA) scanner (Hologic Inc, Bedford, MA); to optimize precision, scans were reviewed (by J.E.A.) and centrally analyzed (in Manchester) by a single operator using APEX 3.1 software. Local quality assurance procedures were monitored centrally, and cross-calibration between scanners was performed by scanning the European Spine Phantom at the start and end of the study (17,18).

Measures from these scans included appendicular lean mass (ALM; the sum of the fat-free mass in the limbs excluding bone mineral content) and whole-body fat mass converted into kilograms. Where data from one arm or leg were missing (*n* = 89 and 61, respectively), data from the other limb were mirrored. A total of 1,636 participants had valid measures for their upper limbs and 1,615 for their lower limbs (with missing data due to the exclusion of measures judged to be affected by knee and hip replacements, metal in the limbs, or the limb falling outside the scan field). Appendicular lean mass index (ALMI) was derived by dividing ALM (kilogram) by height (square meter), which was measured by nurses using standardized protocols as part of the anthropometric assessment conducted at the same time as the DXA measurement. To derive a measure of ALM adjusted for height and fat mass, residuals were generated from sex-specific models in which ALM (kilogram) = β_0_ + β_1_ height (meter) + β_2_ fat mass (kilogram) (7).

Grip strength (ie, muscle force [kilogram]) was assessed by nurses using standardized protocols using an electronic handgrip dynamometer, as previously described in detail elsewhere (13). Three values were recorded for each hand and the highest value was used in analyses. As in other studies, muscle quality (ie, muscle force/mass) was derived by dividing maximum grip strength (kilogram) by upper body ALM (kilogram) (19).

### Body Mass Index

Heights and weights measured by nurses using standardized protocols at ages 15, 36, 43, 53, and 60–64 years and self-reported at 20 and 26 years were used to calculate BMI at each age (weight at specified age [kilogram]/height at specified age^2^ [square meter]). To ensure comparability of analyses across ages and sex, sex-specific *z*-scores of BMI at each age (with a mean of 0 and standard deviation of 1) were then derived.

### Analysis

Four binary outcome variables were created that distinguished between those people in the bottom sex-specific 20% and top 80% of the distributions of ALMI, ALM residuals, grip strength, and muscle quality, among those with data on all four measures. This cut-point was chosen a priori as it enables fair comparisons of results and has been used in other studies (7).

Mean BMI at each age (15 to 60–64 years) was plotted by each of the four binary outcomes stratified by sex. The associations of sex-standardized BMI at each age with each outcome were then formally tested using logistic regression.

In order to test whether there were differential effects of BMI gain in earlier and later adulthood on each of the four main outcomes, the conditional changes in BMI between ages 15–36 and 36 to 60–64 were calculated. We selected 36 years as the midpoint because of the increasing prevalence of obesity in the NSHD, suggesting greater fat mass accrual, from this age onwards. We regressed each BMI measure on the earlier measure(s) for each sex and calculated the residuals (20). These residuals can be interpreted as the change in BMI above or below that expected given earlier BMI. The residuals were standardized to ensure their comparability. Logistic regression models were then run that included the standardized residuals for both intervals of change and each binary outcome, and Wald tests were used to formally compare differences between the two coefficients.

Finally, we examined whether the associations of obesity with muscle accumulated across life by testing the association of each outcome with a variable indicating age first obese, using those who were never obese as the reference group.

In all models, sex interactions and deviations from linearity were formally tested and models were sex stratified where necessary.

### Sensitivity Analyses

Sensitivity analyses were run in which (a) the four main binary outcomes were generated including all available participants (rather than being restricted to the sample with valid data on all four measures), (b) those unable to perform the grip strength tests for health reasons (*n* = 49) were included in the bottom 20%, (c) grip strength was adjusted for height prior to identifying those in the bottom 20% of the distribution, (d) BMI was modeled in units of kilogram per square meter rather than standard deviation scores, and (e) BMI at each age was calculated using height at age 36 years to check that changes in BMI with age were explained by changes in weight rather than changes in height.

## Results

Men and women with low ALMI and ALM residuals had lower mean BMI and whole-body fat mass at ages 60–64 years than those with higher values of these measures, whereas the reverse was found for muscle quality (Table 1). The correlation coefficients between ALM and grip strength were low (Supplementary Table 1).

**Table 1.** Anthropometrics of the Medical Research Council National Survey of Health and Development Study Participants at Age 60–64 Years Stratified By Sex and ALMI, ALM Residuals, Grip Strength, and Muscle Quality (*n* = 1,511; sample includes those with complete data on all four outcome measures)

	Mean (*SD*) unless otherwise specified								
	Total	ALMI		ALM residuals		Grip strength		Muscle quality	
		Bottom 20%	Top 80%	Bottom 20%	Top 80%	Bottom 20%	Top 80%	Bottom 20%	Top 80%
Men									
***N*** ^†^ **(%)**	728 (100)	145 (19.9)	583 (80.1)	145 (19.9)	583 (80.1)	142 (19.5)	586 (80.5)	145 (19.9)	583 (80.1)
Height (m)	1.75 (0.06)	1.76 (0.07)	1.75 (0.06)	1.76 (0.07)	1.75 (0.06)	1.73 (0.06)	1.76 (0.06)*	1.75 (0.06)	1.75 (0.06)
Whole-body fat mass (kg)	25.1 (7.3)	20.8 (4.9)	26.1 (7.4)*	25.2 (7.6)	25.1 (7.2)	24.8 (7.5)	25.1 (7.2)	27.6 (7.9)	24.5 (7.0)*
BMI (kg/m^2^)	27.7 (3.9)	23.8 (2.2)	28.7 (3.6)*	25.8 (3.9)	28.2 (3.8)*	27.3 (4.0)	27.9 (3.9)	29.4 (4.1)	27.3 (3.8)*
Obese (%) (ie, BMI ≥ 30)	26.8%	0%	33.5%*	12.4%	30.4%*	22.5%	27.8%*	40.7%	23.3%*
Women									
***N*** ^†^ **(%)**	783 (100)	156 (19.9)	627 (80.1)	156 (19.9)	627 (80.1)	151 (19.3)	632 (80.7)	156 (19.9)	627 (80.1)
Height (m)	1.62 (0.06)	1.62 (0.06)	1.62 (0.06)	1.63 (0.06)	1.62 (0.06)	1.60 (0.06)	1.63 (0.06)*	1.62 (0.06)	1.62 (0.06)
Whole-body fat mass (kg)	30.1 (9.3)	24.3 (5.8)	31.5 (9.4)*	30.6 (8.1)	29.9 (9.5)	30.2 (9.4)	30.0 (9.3)	33.9 (10.5)	29.1 (8.7)*
BMI (kg/m^2^)	27.5 (5.0)	23.2 (2.6)	28.6 (4.9)*	26.1 (4.0)	27.9 (5.2)*	27.7 (5.5)	27.5 (4.9)	29.9 (5.9)	26.9 (4.6)*
Obese (%) (ie, BMI ≥ 30)	27.8%	0.6%	34.6%*	19.9%	29.8%^**‡**^	27.8%	27.9%	46.8%	23.1%*

*Notes:* ALM = appendicular lean mass; ALMI = appendicular lean mass index; BMI = body mass index.

20% cut-points for ALMI: men < 7.189 kg/m^2^; women: <5.472 kg/m^2^; ALM residuals: men: ≤ −2.08; women: ≤ −1.51.

Grip strength: men < 37.2 kg; women: <20.8 kg.

Muscle quality (grip strength [kilogram]/arm lean mass [kilogram]): men <5.76 kg/kg; women <5.475 kg/kg.

Equations for ALM residuals method: men (*n* = 728) ALM (kilogram) = −22.90 + 24.22 (height [meter]) + 0.20 (whole body fat mass [kilogram]); women (*n* = 783) ALM (kilogram) = −14.59 + 15.97 (height [meter]) + 0.16 (whole body fat mass [kilogram]).

*p ≤ .001 (*p*-values not indicated if *t* test of difference resulted in *p* > .10).

^†^ *N*s vary by covariate due to missing data (all descriptive statistics presented based on maximum available samples).

^‡^001 < *p* < .05.

**Figure 1.**
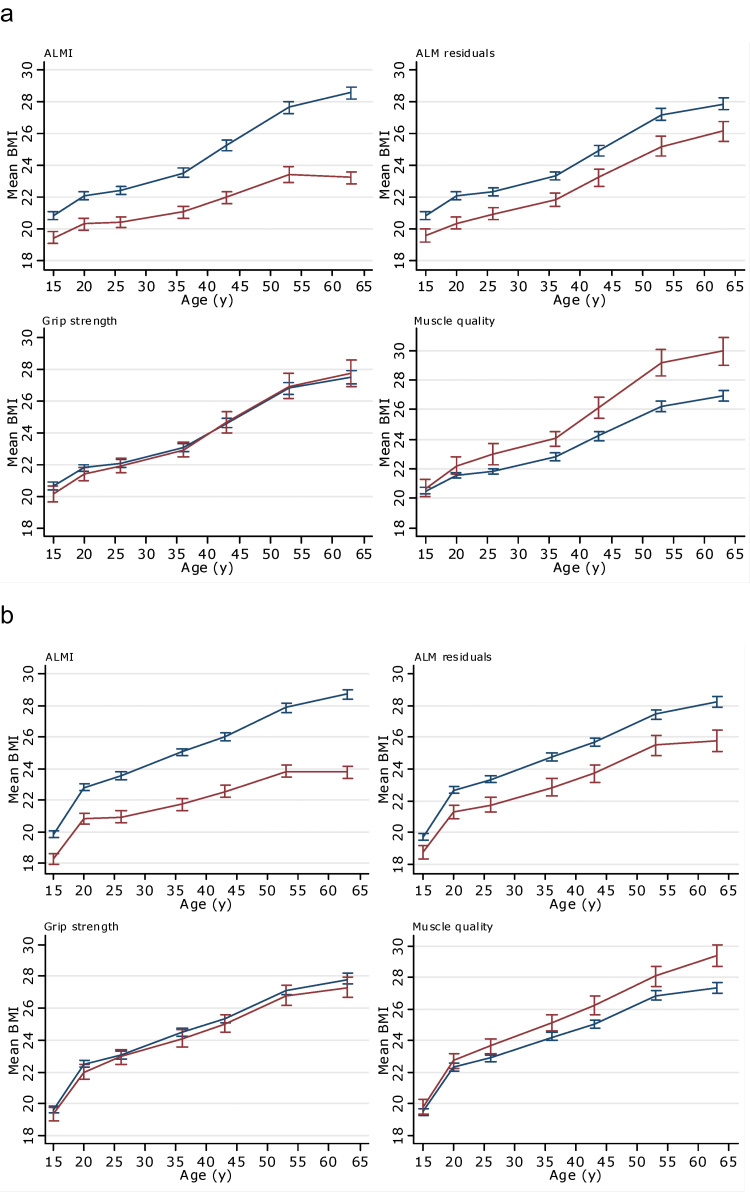
Mean BMI (kilogram per square meter) from age 15 years onwards stratified by ALMI, ALM residuals, grip strength, and muscle quality in (**a**) women and (**b**) men. Blue line indicates those in top 80% of distribution of specified muscle measure, and red line indicates those in bottom 20%. Sample restricted to those with data on all measures of muscle (maximum *n* = 783 women and 728 men). ALMI = appendicular lean mass index (ie, ALM adjusted for height); ALM residuals = appendicular lean mass residuals (ie, ALM adjusted for height and fat mass); muscle quality = grip strength (kilogram)/arm lean mass (kilogram). BMI = body mass index.

Differences in the patterns of the distribution of BMI by each outcome were evident in both sexes (Figure 1a and b). Those with low ALMI and ALM residuals had a lower mean BMI from 15 years onwards when compared with those with higher levels. Conversely, those with low muscle quality had higher mean BMI from 26 years onwards when compared with those with higher muscle quality. No clear differences in BMI by grip strength were observed. When formally tested, these differences in the patterns of association with BMI at each age of assessment (from ages 15 to 60–64 years) were confirmed (Supplementary Table 2).

Greater gains in BMI between ages 15–36 and 36 to 60–64 years were both associated with lower odds of low ALMI and higher odds of low muscle quality at age 60–64 years (Table 2), with no evidence to suggest that the effects of BMI gain in the two periods differed for either outcome. However, greater BMI gain in earlier adulthood was associated with lower odds of low ALM residuals, whereas greater BMI gain in later adulthood was not. BMI gain in neither age period influenced the odds of low grip strength.

**Table 2.** Odds Ratios of Being in the Bottom Sex-specific 20% of the Distribution of ALMI, ALM Residuals, Grip Strength, and Muscle Quality By Conditional BMI Change (in the intervals 15–36 and 36 to 60–64 years; adjusted for sex; *N* = 1,081)

Interval of BMI change	OR (95% CI) of being in the bottom sex-specific 20% of the distribution of the specified measure of muscle per 1 *SD* change in BMI in each interval			
	ALMI	ALM residuals	Grip strength	Muscle quality
15 to 36 y	0.33 (0.27, 0.42)	0.65 (0.55, 0.78)	1.00 (0.85, 1.18)	1.46 (1.23, 1.72)
36 to 60–64 y	0.35 (0.28, 0.43)	0.94 (0.80, 1.10)	1.03 (0.88, 1.21)	1.47 (1.25, 1.74)
*p*-value^*^	.81	.003	.76	.93

*Notes:* ALMI = appendicular lean mass index; ALM residuals = appendicular lean mass residuals; BMI = body mass index; OR = odds ratio.

There was little evidence of sex interaction in any of these models: *p* = .06 for ALMI, .12 for ALM residuals, .23 for grip strength, and .76 for muscle quality; sex-stratified OR of low ALMI associated with change in BMI between 15–36 and 36 to 60–64 y, respectively, were in men: 0.27 (0.19, 0.38), 0.39 (0.29, 0.53); in women: 0.41 (0.30, 0.56), 0.31 (0.23, 0.41).

ORs represent the odds ratio of being in the bottom 20% of the distribution of the specified measure of muscle (compared with the odds for being in the top 80%) per 1 *SD* change in BMI in the specified interval conditional on earlier BMI (ie, 1 *SD* change in the residuals from sex-specific models in which each BMI measure is regressed on the earlier measure(s) (ie, 36 on 15 and 60–64 on 36 and 15).

OR < 1: greater gain in BMI in the specified age interval associated with reduced odds of low levels of the specified measure of muscle.

OR > 1: greater gain in BMI in the specified age interval associated with increased odds of low levels of the specified measure of muscle.

*From Wald test of the difference between the two coefficients.

The association between age at onset of obesity was assessed in relation to muscle quality only; there was insufficient statistical power to study the ALM outcomes as very few participants with low ALM were obese at any age and there was no evidence of associations between BMI and grip strength in previous stages of analyses. Becoming obese at any age across adulthood was associated with increased odds of low muscle quality at age 60–64 years with some suggestion of larger effects with exposure to obesity by age 43 years (Table 3).

**Table 3.** Odds Ratios (95% CIs) of Being in the Bottom 20% of the Distribution of Muscle Quality By Age First Obese (adjusted for sex [test for sex interaction *p* = .57])

Age (y) first obese	*N*	Odds ratio (95% CI)
Never obese	810	1.00
60–64	113	1.85 (1.15, 2.97)
53	134	2.67 (1.76, 4.04)
43	69	3.96 (2.35, 6.65)
26 or 36	56	2.36 (1.28, 4.36)
Test for trend		*p* < .01

There were no substantive differences in findings when each of the different sensitivity analyses were performed (results available on request) and, the latter set of these analyses confirmed that increases in BMI observed between ages 15 and 64 years were attributable to weight gain rather than height loss.

## Discussion

In a large nationally representative study of British men and women, greater gains in BMI from age 15 years onwards were associated with reduced odds of low lean mass in early old age but not with grip strength. Therefore, greater gains in BMI were associated with increased odds of low muscle quality.

Our study is consistent with published cross-sectional findings showing (a) differences in BMI between those with low and higher levels of lean mass that are partially attenuated if ALM is adjusted for fat mass (7,9,21) and (b) inverse associations between fat mass and muscle quality (11,12). Our work extends these previous findings by demonstrating differential effects of BMI from age 15 onwards on muscle mass, strength, and quality.

Our finding of no associations between BMI and grip strength at ages 60–64 years suggests that associations have weakened since age 53 years (13). In a meta-analysis of eight studies, including NSHD at age 53 years, BMI was positively associated with grip strength among men but not women (10). However, these associations were nonlinear, driven by the weaker grip strength of men in the bottom 20% of BMI. In addition, there was some evidence that the association between BMI and grip strength among men was weaker in older cohorts. The older age of NSHD participants in these new analyses and different methods of modeling grip strength may, therefore, explain differences in findings.

Analyses of the Health ABC study have shown cross-sectional associations between higher fat mass and greater muscle strength; however, fat mass was not associated with subsequent declines in strength (12). In a Finnish study of adults aged 55 and older, a negative impact of obesity across adulthood, which was retrospectively assessed, on grip strength was reported (14). However, in this Finnish study, models were adjusted for current weight, and so the findings can be explained by the positive association of height with grip strength (22). These inconsistencies in findings suggest that there may be real differences in the associations of obesity with grip strength by factors such as age, birth cohort, and country, with differences in other factors such as study design and analytical approach potentially introducing artifactual differences.

The finding of differential patterns of association between BMI and muscle mass, strength, and quality highlights the importance of considering these measures of muscle as distinct from each other. The low correlation between our DXA measures of muscle mass and grip strength and only limited overlap between those in the bottom 20% of the distributions of muscle mass, strength, and quality confirm this.

Our finding of positive associations between BMI and lean mass in early old age is consistent with the existence of adaptive physiological responses that result in people with higher fat mass obtaining greater levels of lean mass (23), which maintains support for movement. That BMI gains in earlier adulthood were associated with lower odds of low ALM residuals, but BMI gains in later adulthood were not, suggests that these compensatory mechanisms become less effective with increasing age and greater fat mass accrual, possibly resulting in insufficient muscle mass to support greater fat mass in later life. This finding could also be partly explained by the fact that in this cohort, BMI gain in earlier adulthood may be more likely to reflect accrual of muscle mass, whereas BMI gain in later adulthood is more likely to reflect accrual of fat mass. NSHD had relatively low mean BMI in earlier adulthood, so this explanation may be cohort specific. In populations born more recently, which have experienced greater fat mass accrual from younger ages, sarcopenic obesity is thus likely to be an increasing public health concern.

The detrimental effect of high BMI on muscle quality may be due to greater levels of fat infiltration of muscle among those of higher BMI or to changes in endocrine function, insulin resistance, and inflammation associated with higher BMI and poorer muscle quality (24).

Major strengths of this study are its use of multiple measures of muscle and fat at age 60–64 years and prospective measurement of BMI over more than 50 years of follow up.

Limitations of our analyses include the lack of data on lower body strength and the availability of DXA measures of body composition at only the final assessment, thus BMI had to be used to indicate adiposity in earlier adulthood. BMI at 20 and 26 years was based on self-reported data on height and weight, which are expected to be less accurate than nurse measures. For this reason, we chose to include BMI at age 15 years as our first measure. Although our study population was selected to be nationally representative at baseline (and remains so in many respects (16)) losses to follow up due to death and nonparticipation have occurred. Our analyses were restricted to those with a DXA assessment who attended a clinic. These participants were in better health and less likely to be obese than those who were visited at home (16); the exclusion of home visit participants may thus have introduced bias. However, when analyses for grip strength were rerun including those participants who had undergone a home visit, there were no changes in findings. In addition, inclusion in sensitivity analyses of those people unable to perform the grip strength assessment for health reasons did not alter findings suggesting that any bias introduced due to the necessary exclusion of some participants was likely to be minimal.

Muscle strength and quality are often found to be more strongly associated with functional outcomes and mortality than muscle mass (8,25–27). Our finding of cumulative associations of obesity with low muscle quality is, therefore, important especially when considering the likely impact of the increasing prevalence of obesity on the health and physical capability of future generations of older people.

Differences in the patterns of association between BMI from age 15 years onwards and different characteristics of muscle suggest that findings from different epidemiological studies, which have used different characteristics of muscle to define sarcopenia or which have differences in obesity prevalence, are unlikely to be fully comparable. Importantly, they also suggest that combining a set of different measures of muscle in one score, which may be important for clinical prognosis, could disguise effects of risk factors when exploring the underlying etiology.

Our finding of strong positive relationships between BMI and ALM, even after adjustment for fat mass, suggests that even if obese people are losing more lean mass than people of normal weight, this is unlikely to be detected in cross-sectional assessments as they will still have, on average, higher absolute levels. Longitudinal assessment of rate of loss of muscle mass, especially in populations with high prevalence of overweight and obesity, is therefore likely to be important when identifying those at greatest risk of sarcopenia.

## Supplementary Material

Supplementary material can be found at: http://biomedgerontology.oxfordjournals.org/

## Funding

This work, the National Survey of Health and Development, and R.C., R.H., D.B., A.A.S., K.A.W., and D.K. are supported by the UK Medical Research Council (MC_UU_12019/4 and U105960371).

## Conflict of Interest

None declared.

## Supplementary Material

Supplementary Data
