# Supplementary material for: Body Mass Index From Age 15 Years Onwards and Muscle Mass, Strength, and Quality in Early Old Age: Findings From the MRC National Survey of Health and Development
Source: J Gerontol A Biol Sci Med Sci. 2014 Mar 28;69(10):1253–9. doi: 10.1093/gerona/glu039 (PMC4158414; doi:10.1093/gerona/glu039)
Supplement: Supplementary Data [file supp_69_10_1253__index.html]

Body Mass Index From Age 15 Years Onwards and Muscle Mass, Strength, and Quality in Early Old Age: Findings From the MRC National Survey of Health and Development — Body Mass Index From Age 15 Years Onwards and Muscle Mass, Strength, and Quality in Early Old Age: Findings From the MRC National Survey of Health and Development — Supplementary Data 

# Body Mass Index From Age 15 Years Onwards and Muscle Mass, Strength, and Quality in Early Old Age: Findings From the MRC National Survey of Health and Development

## Supplementary Data

Data files

**Files in this Data Supplement:**

- Supplementary Data - Supplementary Data
