# Supplementary material for: Body Mass Index From Age 15 Years Onwards and Muscle Mass, Strength, and Quality in Early Old Age: Findings From the MRC National Survey of Health and Development
Source: J Gerontol A Biol Sci Med Sci. 2014 Mar 28;69(10):1253–9. doi: 10.1093/gerona/glu039 (PMC4158414; doi:10.1093/gerona/glu039)
Supplement: Supplementary Data [file supp_glu039_Supplementary_results_JGerontol_REVISED.docx]

**Supplementary table 1: Correlation coefficients between different measures of body composition and muscle at 60-64 years**

**[restricted to sample with complete data on 4 main measures of muscle (N=1511)]**

|  | **ALMI** | **ALM Residuals** | **Grip strength** | **Muscle quality** | **Whole body fat mass** | **BMI** | **Height** |
| --- | --- | --- | --- | --- | --- | --- | --- |
| **ALMI** | 1.00 | 0.75 | 0.10 | -0.34 | 0.65 | 0.81 | -0.01 |
| **ALM Residuals** | 0.87 | 1.00 | 0.19 | -0.21 | 0.00 | 0.24 | 0.00 |
| **Grip strength** | 0.27 | 0.32 | 1.00 | 0.81 | -0.03 | -0.04 | 0.24 |
| **Muscle quality** | -0.24 | -0.14 | 0.75 | 1.00 | -0.26 | -0.31 | 0.005 |
| **Whole body fat mass** | 0.49 | 0.00 | 0.03 | -0.24 | 1.00 | 0.93 | 0.13 |
| **BMI** | 0.78 | 0.38 | 0.07 | -0.29 | 0.87 | 1.00 | -0.10 |
| **Height** | -0.002 | 0.00 | 0.27 | -0.01 | 0.16 | -0.09 | 1.00 |

ALMI = appendicular lean mass index (i.e. ALM adjusted for height)

ALM residuals = appendicular lean mass residuals (i.e. ALM adjusted for height and fat mass)

BMI = body mass index

**Men (n=728) Women (n=783)**

**Supplementary table 2: Odds ratios (95% CIs) for being in the bottom 20% of the distribution of the specified measure of muscle at age 60-64 years per 1SD difference in BMI at each age across adulthood (adjusted for sex (unless evidence of sex interaction found))**

| **BMI at age:** | **ALMI** |  | **ALM residuals** |  | **Grip strength** |  | **Muscle quality** |
| --- | --- | --- | --- | --- | --- | --- | --- |
| 15y (n=1173) | 0.51 (0.43, 0.60) |  | 0.60 (0.51, 0.70) |  | 0.86 (0.74, 1.00) |  | 1.09 (0.94, 1.27) |
| 20y (n=1243) | 0.40 (0.33, 0.47) |  | 0.48 (0.41, 0.56) |  | 0.81 (0.70, 0.93) |  | 1.18 (1.02, 1.37) |
| 26y (n=1331) | 0.33 (0.27, 0.39) |  | 0.52 (0.45, 0.61) |  | 0.94 (0.82, 1.08) |  | 1.40 (1.21, 1.61) |
| 36y (n=1371)  (654 men, 717 women) | M: 0.22 (0.16, 0.29)  W: 0.33 (0.26, 0.43)  p^*^=0.03 |  | 0.50 (0.43, 0.59) |  | 0.91 (0.79, 1.06) |  | 1.50 (1.29, 1.75) |
| 43y (n=1425)  (685 men, 740 women) | M: 0.18 (0.13, 0.25)  W: 0.29 (0.22, 0.38)  p^*^=0.03 |  | 0.51 (0.44, 0.60) |  | 0.95 (0.82, 1.09) |  | 1.61 (1.39, 1.86) |
| 53y (n=1439)  (683 men, 812 women) | M: 0.16 (0.12, 0.23)  W: 0.26 (0.20, 0.34)  p^*^=0.03 |  | 0.56 (0.48, 0.65) |  | 0.95 (0.82, 1.09) |  | 1.68 (1.45, 1.95) |
| 60-64y (n=1511)  (728 men, 783 women) | 0.08 (0.06, 0.11) |  | M: 0.46 (0.36, 0.58)  W: 0.65 (0.52, 0.81)  p^*^=0.03 |  | 0.96 (0.83, 1.10) |  | 1.77 (1.55, 2.02) |

M = Men; W = Women

* p-value from formal test of sex interaction

Note: Sample includes those with complete data on all four measures of muscle, maximum N=1511 but N varies at different ages of BMI assessment due to variation in the amount of missing data on BMI at each age

ALMI = appendicular lean mass index (i.e. ALM adjusted for height)

ALM residuals = appendicular lean mass residuals (i.e. ALM adjusted for height and fat mass)

Values of 1SD of BMI (kg/m^2^) in men (M) and women (W) at each age examined are as follows: 15y: M=2.43, W=3.02; 20y: M=2.47, W=2.90; 26y: M=2.83, W=3.25; 36y: M=3.25, W=4.06; 43y: M=3.52, W=4.78; 53y: M=4.03, W=5.45; 60-64y: M=4.10, W=5.28
